# In-yeast reconstruction of the African swine fever virus genome isolated from clinical samples

**DOI:** 10.1016/j.xpro.2021.100803

**Published:** 2021-09-08

**Authors:** Fabien Labroussaa, Kemal Mehinagic, Valentina Cippa, Matthias Liniger, Hatice Akarsu, Nicolas Ruggli, Joerg Jores

**Affiliations:** 1 Institute of Veterinary Bacteriology, University of Bern, Bern 3001, Switzerland; 2 Department of Infectious Diseases and Pathobiology (DIP), Vetsuisse Faculty, University of Bern, Bern 3001, Switzerland; 3 Institute of Virology and Immunology (IVI), Sensemattstrasse 293, Mittelhäusern 3147, Switzerland; 4 Graduate School for Cellular and Biomedical Sciences, University of Bern, Bern 3001, Switzerland

**Keywords:** Health Sciences, Genomics, Sequencing, Microbiology, Molecular Biology, Systems biology, Biotechnology and bioengineering

## Abstract

This protocol describes a synthetic genomics pipeline to clone and engineer the entire 190-kbp genome of the African swine fever virus (ASFV) genotype II in yeast using transformation-associated recombination cloning. The viral genome was cloned using DNA directly extracted from a clinical sample. In addition, the precise deletion of a non-essential gene and its replacement by a synthetic reporter gene cassette are presented. This protocol is applicable to other ASFV genotypes and other large DNA viruses.

## Highlights

• Use of TAR for the individual cloning of five ASFV sub-genomic fragments in yeast
• Chemical synthesis of both 5′ and 3′ ITR genomic regions
• Replacement of the C962R gene by reporter genes (eGFP or secNLuc)

## Graphical abstract

**Figure:**
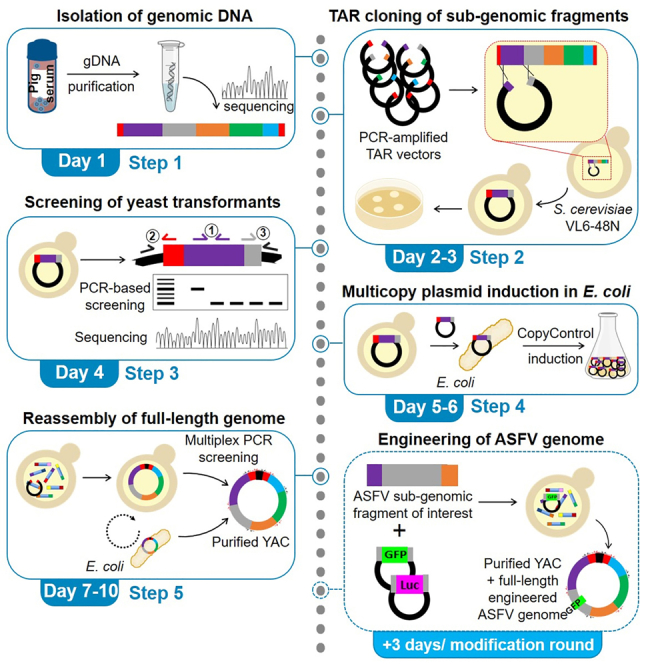

## Before you begin

ASFV causes a devastating hemorrhagic disease in pigs with a mortality often reaching up to 100% (Sánchez-Cordón et al., 2018). ASFV is rapidly spreading throughout Europe and Asia, already responsible for the loss of approximatively a third of the world’s pig population. The development of effective treatments and vaccines are hindered due to knowledge gaps regarding the biology of the virus (Sánchez et al., 2019).

ASFV is a large DNA virus belonging to the *Asfarviridae* family with a genome size ranging between 170 and 190 kbp (Dixon et al., 2013), a size problematic to be stably maintained in *E. coli*-based systems commonly used to generate and mutagenize molecular clones. The generation of defined ASFV mutants still relies on homologous recombination methods mainly involving eukaryotic cells such as swine macrophages (Borca et al., 2021; Gallardo et al., 2018; Monteagudo et al., 2017; O’Donnell et al., 2015), although the use of CRISPR-Cas9 technology was recently implemented (Borca et al., 2018). In addition, ASFV replication in cell culture has been reported to result in adaptive single nucleotide mutations, genome deletions and rearrangements associated with phenotype changes (Borca et al., 2021; Krug et al., 2015).

Here we describe a yeast-based protocol for the cloning and engineering of an ASFV isolate belonging to the genotype II, the genotype responsible for the current pandemic in Europe and Asia. This pipeline relies on the transformation-associated recombination (TAR) cloning strategy, which was originally developed for the selective isolation and maintenance of large eukaryotic DNA regions in *Saccharomyces cerevisiae* (Larionov et al., 1996). The TAR cloning method uses the superior capacities of the yeast to perform *in vivo* homologous recombination and diverts it in order to recombine several overlapping DNA fragments together. This system was adopted to reconstruct the genome of the first synthetic mycoplasma cell (Syn 1.0; Gibson et al., 2010), but was also applied to other large DNA viruses such as the Herpes simplex virus type 1 (HSV-1; Oldfield et al. 2017) and, more recently, to RNA viruses including the SARS-CoV-2 (Thao et al., 2020).

This protocol includes all the necessary steps from the extraction of ASFV genomic DNA from clinical samples up to the reconstruction of full-length ASFV genomes, natural or engineered, in the yeast *S. cerevisiae*. This protocol does not include a rescue system, which has to be developed and adapted separately for the different viruses to be tackled.

### Biosafety

In Switzerland, ASFV is a risk group 4 pathogen and hence all work involving live virus was carried out in the BSL-3Ag containment facility of the Institute of Virology and Immunology in Mittelhäusern (Switzerland). After inactivation, the sera from ASFV-infected pigs were transferred out of the BSL-3Ag environment to standard BSL-2 laboratories, where the extraction of the viral genomic DNA and all the following steps of this protocol were performed. Animal experiments were conducted at the IVI in compliance with the animal welfare regulation of Switzerland under the cantonal license BE18/2019.

### Design of the ASFV genome reconstruction

**Timing: ∼2 h (user dependent)**

**Figure 1:**
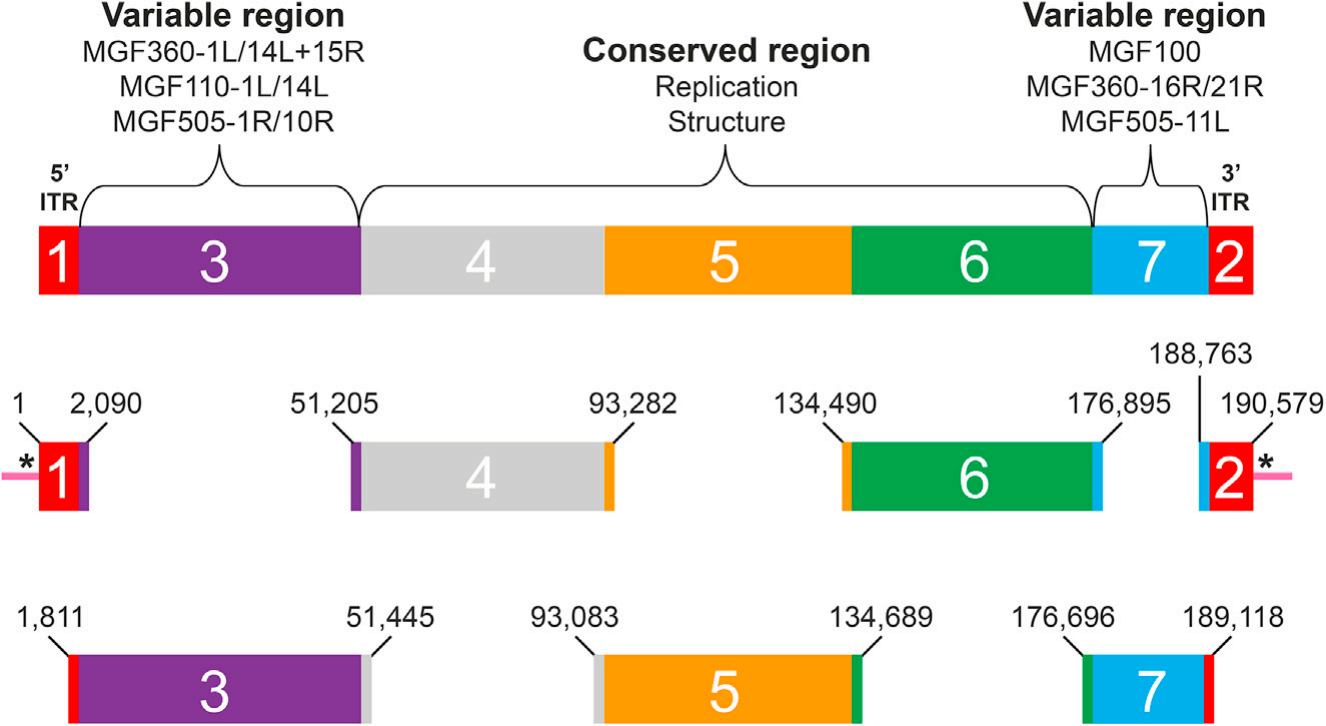
TAR cloning design for the reconstruction of the ASFV genome The genome was divided into seven sub-genomic fragments. Fragments 1 and 2, encompassing highly repetitive ITRs, were chemically-synthetized. Both contain an artificial 50-bp sequence stretch (in pink) at their 5′ and 3′-ends, respectively. These 50-bp stretches will be clipped off via restriction endonucleases indicated by the asterisks after full-length genome assembly. Positions indicated for each of the seven sub-genomic fragments correspond to the ASFV nucleotide sequence as provided in GenBank.

The *in silico* design used in this protocol involves the fragmentation of the ASFV genome into seven sub-genomic fragments as presented in Figure 1.

1. The 5′ and 3′-ends of the ASFV genome, consisting of inverted-terminal-repeats (ITRs), are chemically-synthetized (GenScript) in order to prevent any illegitimate recombination events during the final reassembly in yeast.
  a. Both synthetic fragments, named Fragment 1 and 2 respectively, are ligated into the vector pUC57 using the unique *EcoRV* restriction site, clone in *E. coli* and sequence-verified.
  b. They contain two *SmaI* restriction sites at their 5′ and 3‘-ends, allowing their excision from the pUC57 vector.
  c. Fragment 1 is a 2,090-bp dsDNA fragment consisting of a 50-bp unique region (randomly designed for site-directed TAR cloning purposes), followed by an *I-SceI* restriction site and by the first 2,010 nucleotides of the ASFV genome.
  d. Fragment 2 consists of the last 1,736 nucleotides of the ASFV genome followed by an *I-SceI* restriction site and by another 50-bp unique region (also randomly designed for TAR cloning).
  e. Their corresponding sequences are provided as supplementary material.
2. The other five sub-genomic fragments ranging in size from 12.4 to 49.6 kbp were isolated using the TAR cloning method. Their corresponding start and end positions are displayed in Figure 1.
  a. Fragment 4, 5 and 6 mainly contain genes encoding proteins involved in virus replication and structure.
  b. Fragments 3 and 7 encompass genes included in multi-gene family (MGF) 100, 110, 360 and 505, most of which still have an unknown function (Dixon et al., 2013; Zhu et al., 2020).
  c. Alternative designs consisting of different numbers of sub-genomic fragments with different 5’ and/or 3′-ends are generally possible, but have not been investigated here. As a general rule of thumb, fragmentation designs aiming for a higher number of shorter sub-genomic fragments require more experimental time (both in term of design and isolation of these genomic intermediates) but will ultimately result in more flexible assemblies.

### Design of the primers

**Timing: 1 h–2 h (user dependent)**

Primers used throughout the protocol are listed in the key resources table (here).

3. In addition to their 3′-binding regions with the pCC1BAC-Ura3(-His3) TAR vectors, all the primers used for TAR cloning contain a 50-bp region (in bold) complementary to the 5′-end (reverse primers) or the 3′-end (forward primers) of the ASFV sub-genomic fragment of interest. Such overlaps (also called hooks) are required for the specific and legitimate homologous recombination in yeast.
4. The presence of an *I-SceI* restriction site (underlined) allows the linearization of the ASFV sub-genomic fragments after their isolation and purification as yeast artificial chromosomes (YACs).
5. Due to their length, all primers used for TAR cloning are purified on polyacrylamide gel electrophoresis (PAGE purified) (Microsynth, Balgach, Switzerland). All other primers used for the PCR-based screening and multiplex PCRs of the different yeast clones were only desalted after synthesis.

## Key resources table

**Table:** 

REAGENT or RESOURCE	SOURCE	IDENTIFIER
**Bacterial and virus strains**		

Stellar™ competent *E. coli*	Takara Bio	636763
TransforMax™ Epi300™ Electrocompetent *E. coli*	Epicentre/Lucigen	EC300110
ASFV isolate Georgia 2007	LMA, Tbilisi (Georgia)	This work

**Experimental models: Organisms/strains**		

*S. cerevisiae* VL6-48N	JCVI	Noskov et al., 2002

**Recombinant DNA**		

pCC1BAC-His3/Ura3	JCVI	Gibson et al., 2010
pUC57-P_Pol-secNluc _C962R-int	GenScript	This work
pUC57-P_Pol-eGFP_C962R-int	GenScript	This work
pUC57-Fragment1	GenScript	This work
pUC57-Fragment2	GenScript	This work

**Oligonucleotides**		

TAR cloning (ASFV WT) ASFTARhook-1R: **gtctagctagcatctagtatgaatcg** **gattcatggcatgcatgcaatcgt**TAGGGATAACAGGGTAAT GTCGTGACTGGGAAAACCCTG	This paper	N/A
ASFTARhook-2F: **taacctgacattgcgatccgtaca** **attgcatgctggaactacctgatcag**TAGGGATAACAGGGTAAT GATCCTCTAGAGTCGACCTGCAG	This paper	N/A
ASFTARhook-3R: **tgttaatatatgaagaatcattgt** **ttcacatctatggggtaaacatttag**TAGGGATAACAGGGTAAT GTCGTGACTGGGAAAACCCTG	This paper	N/A
ASFTARhook-3F: **cagaacatttagataattgaga** **gattactttccatacttgttaagctttt**TAGGGATAACAGGGTAAT GATCCTCTAGAGTCGACCTGCAG	This paper	N/A
ASFTARhook-4R: **atagatactcaaaagacagt** **cgttcatgacgtaagattgtcgctttaatc**TAGGGATAACAGGGTAAT GTCGTGACTGGGAAAACCCTG	This paper	N/A
ASFTARhook-4F: **caggctgtgaagccagaaggcctcctgaccttgatga** **aggtcgtacatga**TAGGGATAACAGGGTAAT GATCCTCTAGAGTCGACCTGCAG	This paper	N/A
ASFTARhook-5R: **ttagtaaacagccttcggagcacg** **aactgttcatcgtatttaaaaaataa**TAGGGATAACAGGGTAAT GTCGTGACTGGGAAAACCCTG	This paper	N/A
ASFTARhook-5F: **ttcagcgtctagcagcggtttcagctt** **ggcaagatgcgctgagtggtagt**TAGGGATAACAGGGTAAT GATCCTCTAGAGTCGACCTGCAG	This paper	N/A
ASFTARhook-6R: **cccgtgcggtaggttttcgtgaa** **ccgataaatgttttagaaatcatttaa**TAGGGATAACAGGGTAAT GTCGTGACTGGGAAAACCCTG	This paper	N/A
ASFTARhook-6F: **agcaatatcctcttctatctcgca** **atcctcctcctccatttccatagtgt**TAGGGATAACAGGGTAAT GATCCTCTAGAGTCGACCTGCAG	This paper	N/A
ASFTARhook-7R: **cggaatcttcatccgacgatgagtcc** **tattcactttatgatagtttctaa**TAGGGATAACAGGGTAAT GTCGTGACTGGGAAAACCCTG	This paper	N/A
ASFTARhook-7F: **ctctctgatggtgacaaatctccgataggaatatatg** **acgtaacataatt**TAGGGATAACAGGGTAAT GATCCTCTAGAGTCGACCTGCAG	This paper	N/A
TAR cloning (Fragment 4ΔC962R) ASFTARhook-4R: **atagatactcaaaagacagtcg** **ttcatgacgtaagattgtcgctttaatc**TAGGGATAACAGGGTAAT GTCGTGACTGGGAAAACCCTG	This paper	N/A
ASFTARhook-4F2: **cattaggacctctcccgcccatttaaatttttagtttctacaataataaa** TAGGGATAACAGGGTAATGATCCTCTAGAGTCGACCTGCAG	This paper	N/A
Screening of yeast transformants (internal fragments) ASFVint-3F: ccttagtagcggcagatacc	This paper	N/A
ASFVint-3R: atggacaggtttcaatgctcg	This paper	N/A
ASFVint-4F: tcgcatttcgtgttgaaatacg	This paper	N/A
ASFVint-4R: gcattcctgcctattaatgtgc	This paper	N/A
ASFVint-5F: gttcaagtggtggaggctc	This paper	N/A
ASFVint-5R: agccttccaacgtgttgttgc	This paper	N/A
ASFVint-6F: gagtgcacgtatcagattacg	This paper	N/A
ASFVint-6R: tcgtggtgttcaaggtaatcg	This paper	N/A
ASFVint-7F: gcattaatgaaagctgtacagg	This paper	N/A
ASFVint-7R: gttgaagtccatgaatctctgg	This paper	N/A
Screening of yeast transformants (junctions) pCC1jct-F1: ccattcagctgcgcaactg	This paper	N/A
ASFVFg3-R: tataagcttactgaagccatcc	This paper	N/A
ASFVFg3-F: ctgattaaagcgacaatcttacg	This paper	N/A
pCC1jct-R1: cttccatgtcggcagaatgc	This paper	N/A
ASFVFg4-R: ctaccaaaacctctctacatgc	This paper	N/A
ASFVFg4-F: ccaactccttagggaatatcc	This paper	N/A
ASFVFg5-R: cgatatggacgatgtccagc	This paper	N/A
ASFVFg5-F: tttcggcatatccagcctcc	This paper	N/A
ASFVFg6-R: ctccgagctgcacttttacg	This paper	N/A
ASFVFg6-F: agtattattagaaatggctgtcg	This paper	N/A
ASFVFg7-R: agagattctcctgttattgtgg	This paper	N/A
ASFVFg7-F: tgacctgtagtacgtatgatgg	This paper	N/A
Screening of final ASFV constructs (multiplex PCR for junctions) Multiplex PCR 1 pCC1jct-F1: ccattcagctgcgcaactg	This paper	N/A
ASFVFg1-R: tgaatcggattcatggcatgc	This paper	N/A
ASFVFg3-F2: gttggcaacaatccacagacg	This paper	N/A
ASFVFg4-R2: cagtctttacaggaaacatgg	This paper	N/A
ASFVFg4-F: ccaactccttagggaatatcc	This paper	N/A
ASFVFg5-R: cgatatggacgatgtccagc	This paper	N/A
ASFVFg2-F: tgacattgcgatccgtacaattgc	This paper	N/A
pCC1jct-R1: cttccatgtcggcagaatgc	This paper	N/A
Multiplex PCR 2 ASFVFg1-F: taaaagagaagagtctggactg	This paper	N/A
ASFVFg3-R: tataagcttactgaagccatcc	This paper	N/A
ASFVFg5-F: tttcggcatatccagcctcc	This paper	N/A
ASFVFg6-R: ctccgagctgcacttttacg	This paper	N/A
ASFVFg6-F: agtattattagaaatggctgtcg	This paper	N/A
ASFVFg7-R: agagattctcctgttattgtgg	This paper	N/A
ASFVFg7-F: tgacctgtagtacgtatgatgg	This paper	N/A
ASFVFg2-R: acgtatagttgataaaagtgctgg	This paper	N/A
Screening for eGFP and secNluc reporters integration ASFVreporter-F: gattataaagagtaactcgtagagg	This paper	N/A
ASFVreporter-R: acatgttacgtacagttcacttcc	This paper	N/A

**Chemicals, peptides, and recombinant proteins**		

YPDA	Takara Bio	630306
SD broth (with 2% glucose)	Formedium	CSM0205
-His single Drop-out	Formedium	DCS0071
-Ura single Drop-out	Formedium	DCS0161
Minimal SD Agar Base	Takara Bio	630412
Difco LB Agar, Lennox	DB	240110
Dulbecco’s Phosphate Buffered Saline (10**×**), no calcium, no magnesium	Thermo Fischer	14200067
Lithium acetate dihydrate	Sigma-Aldrich	L4158
ssDNA sodium salt	Sigma-Aldrich	D1626
PEG3350	Sigma-Aldrich	P4338-500G
Chelex 100 sodium form	Sigma-Aldrich	95577-100G-F
Glass beads, acid washed	Sigma-Aldrich	G8772-100G
PrimeSTAR GXL DNA polymerase	Takara Bio	R050B
GoTaq G2 Green Master Mix	Promega	M7823
Multiplex PCR Kit	QIAGEN	206143
Zymolyase®-100T	AMSBIO	120493-1
Glycoblue™ Coprecipitant (15 mg/mL)	Thermo Fischer	AM9516
Ribonucleic acid, transfer from baker's yeast	Sigma	R5636-1ML
Sodium acetate 3M, pH5.2	Thermo Fischer	R1181
RedSafe™ Nucleic Acid Staining Solution	iNtRON	21141
*Dpn*I	NEB	R0176L
*Sma*I	NEB	R0141L
I-*Sce*I	NEB	R0694L
CopyControl™ Induction solution	Lucigen	CCIS125

**Critical commercial assays**		

MagAttract HMW DNA Kit	QIAGEN	67563
QIAprep Spin Miniprep Kit	QIAGEN	27106
QIAGEN Plasmid Midi Kit	QIAGEN	12143
QIAGEN Large-Construct Kit	QIAGEN	12462
High Pure PCR Product Purification Kit	Merck	11732668001
QIAquick Gel Extraction Kit	QIAGEN	28704

**Other**		

Optima™ L-90K Ultracentrifuge with SW41Ti swinging-bucket rotor	Beckman Coulter	N/A
Biospectrophotometer	Eppendorf	6135000009
Lab Armor Bead Bath	LabArmor	M706
Thermomixer C	Eppendorf	5382000015
UltraSlim LED Transilluminator	MaestroGen	SLB-01W
Ultra-Clear Centrifuge Tubes (14 **×** 89 mm, 13.2 mL)	Beckman Coulter	344059
Gene Pulser Xcell™ Electroporation System	Bio-Rad	165–2660/68
Roti®-Store yeast cryo vials	Carl Roth	X983.1
Roti®-Store yeast cryo vials (for *E. coli*)	Carl Roth	P730.1
Gene Pulser®/MicroPulser™ Electroporation Cuvettes, 0.1 cm gap	Bio-Rad	1652089
X-tracta Gel Extractor Tool	Sigma-Aldrich	Z722390
Baffled flasks ROTILABO®, straight neck, 500 mL or 2 L	Carl Roth	LY96.1 or LY98.1

**Software and algorithms**		

Benchling	N/A	https://benchling.com

## Materials and equipment

**Table:** 

Zymolyase solution		
Reagents	Final concentration	Amount
Zymolyase-100T	10 mg/mL	200 mg
Glycerol (50% v/v)	25% (v/v)	10 mL
Tris-HCl (1M, pH7.5)	50 mM	1 mL
ddH_2_O	n/a	9 mL
**Total**	**n/a**	**20 mL**

Solution can be stored in 0.5 mL-aliquots at −20°C up to 1 year. Avoid excessive freezing/thawing cycles.

**Table:** 

SPEM solution		
Reagents	Final concentration	Amount
Na_2_HPO_4_.2H_2_O	7.75 mM	0.69 g
NaH_2_PO_4_.2H_2_O	2.31 mM	0.18 g
EDTA (0,5M, pH7,5)	10 mM	10 mL
Sorbitol	1 M	91 g
ddH_2_O	n/a	Up to 500 mL
Sterilized on 0.22 μm filter		
**Total**	**n/a**	**500 mL**

Store at 20°C–25°C up to 1 year.

**Table:** 

STC solution		
Reagents	Final concentration	Amount
Sorbitol	1 M	18.2 g
Tris (1M, pH7.5)	10 mM	1 mL
CaCl_2_ (1M)	10 mM	1 mL
ddH_2_O	n/a	Up to 100 mL
Sterilized on 0.22 μm filter		
**Total**	**n/a**	**100 mL**

Store at 20°C–25°C for up to 3 months.

**Table:** 

SOS solution		
Reagents	Final concentration	Amount
Sorbitol	1 M	9.1 g
Yeast extract	n/a	0.125 g
Bacto peptone	n/a	0.25 g
CaCl_2_ (1M)	0.6 μM	300 μL
ddH_2_O	n/a	Up to 50 mL
Sterilized on 0.22 μm filter		
**Total**	**n/a**	**50 mL**

Store at 20°C–25°C up to 1 year.

**Table:** 

Sorbitol plates		
Reagents	Final concentration	Amount
Sorbitol	1 M	91 g
SD Base Agar	n/a	23.35 g
-His (or –Ura)	n/a	0.35 g
ddH_2_O	n/a	Up to 500 mL
Adjust the pH to 5.8 and autoclave 121°C 15min		
**Total**	**25 mL/plate**	**500 mL**

Store upside down at 4°C up to 2/3 weeks.

**Table:** 

PEG solution		
Reagents	Final concentration	Amount
PEG 8000	n/a	10 g
Tris-HCl (1M, pH7.5)	10 mM	500 μL
CaCl_2_ 1M	10 mM	500 μL
ddH_2_O	n/a	Up to 50 mL
Adjust the pH to 7.5 and sterilized on 0.22 μm filter		
**Total**	**n/a**	**50 mL**

Prepare fresh for every transformation and place at 20°C–25°C during the experiment.

**Table:** 

TOP agar		
Reagents	Final concentration	Amount
Sorbitol	1 M	45.5 g
SD base	n/a	6.72 g
-His (or -Ura)	n/a	0.2 g
Bacto agar	n/a	7.5 g
ddH_2_O	n/a	Up to 250 mL
Adjust the pH to 5.8 and autoclave 121°C 15min		
**Total**	**12.5 mL /plate**	**250 mL**

Prepare fresh on the morning of each transformation and place at 55°C in a water/bead bath until needed.

**Table:** 

SD-Ura (or-His) plates		
Reagents	Final concentration	Amount
SD Base Agar	n/a	46.7 g
-His (or -Ura)	n/a	0.77 g
ddH_2_O	n/a	Up to 1 L
Adjust the pH to 5.8 and autoclave 121°C 15min		

Store upside down at 4°C up to 2/3 weeks.

**Table:** 

LB-chl^R^_12.5_ plates		
Reagents	Final concentration	Amount
LB Agar	n/a	35 g
Chloramphenicol (50 mg/mL)	12.5 μg/mL	250 μL
ddH_2_O	n/a	Up to 1 L

Store upside down at 4°C up to 2/3 weeks.

## Step-by-step method details

### Concentration of ASFV particles and isolation of high molecular weight viral genomic DNA

**Timing: 2 h**

This step describes the concentration of viral particles from the serum of domestic pigs experimentally infected experimentally with ASFV via ultracentrifugation and the subsequent isolation of high molecular weight viral DNA (vDNA). Organ material from a field case of ASF in Western Georgia in June 2007 was obtained from Tinatin Onashvili and Cezar Machitidze, Laboratory of the Ministry of Agriculture (LMA), Tbilisi, Georgia. Spleen homogenate was used to infect six 6-week-old male specific pathogen-free Large White pigs by oro-nasal (three pigs) or intramuscular (three pigs) application. Sera were collected five days after infection for further processing in this protocol. On the fifth day after infection, the serum of ASFV- infected pigs contained ∼10^8^ ASFV genome equivalents and was processed as described below. The same protocol can be applied for isolation of vDNA from cell-culture supernatants containing viral particles.

1. Form a sucrose cushion by adding 2 mL of a 20% sucrose solution in a 13.2 mL ultra-clear centrifuge tube (Beckman Coulter, see key resources table)
2. Overlay the sucrose with 7 mL of ASFV-infected pig serum by gently pipetting down the side of the tube
3. Carefully add phosphate buffer saline (PBS) (see key resources table) up to 2–3 mm from the top of the tube.
4. Centrifuge at 50,000 **×** *g* for 90 min.

***Note:*** Familiarize yourself with the user manual and safety instructions before using the rotor and operating the ultracentrifuge.

5. Carefully discard the supernatant by pipetting without disturbing the pellet. Usually a discrete pale-white pellet is visible.
6. Resuspend the pellet in 200 μL of PBS.
7. Extract the virus DNA using the MagAttract HMW DNA Kit (Qiagen) according to manufacturer’s instructions (see here).

### Transformation-associated recombination (TAR) cloning of ASFV sub-genomic fragments

**Timing: 1 day (+ 2 days of incubation after yeast transformation)**

This section describes all the necessary steps required for the individual isolation of the different ASFV sub-genomic fragments in yeast. It encompasses the generation of the different TAR vectors carrying the appropriate hook sequences up to their individual co-transformation in yeast spheroplasts along with the previously isolated high-quality ASFV genomic DNA. Each of the five sub-genomic fragments has to be isolated individually but all the transformations can be performed in parallel.

8. Insertion of the overlapping regions (hooks) and I-*Sce*I restrictions sites in the pCC1BAC-Ura3 TAR vector
  a. pCC1BAC-Ura3 plasmid (10 ng/μL) was used as DNA template in the following PCR reaction.

**Table:** 

Reagents	Final concentration	Amount (for one reaction)
ddH_2_O	n/a	32 μL
5**×** PrimeSTAR DNA Buffer	1**×**	10 μL
dNTP mixture (2.5 mM each)	200 μM each	4 μL
Forward primer (10 μM)	0.2 μM	1 μL
Reverse primer (10 μM)	0.2 μM	1 μL
PrimeSTAR GXL DNA polymerase	1.25 U	1 μL
DNA template (TAR vector)	10 ng	1 μL
    **Table:** 

PCR cycling conditions			
Steps	Temperature (°C)	Time	Cycles
Initial denaturation	98	1 min	1
Denaturation	98	10 s	30
Annealing	52	15 s	
Extension	68	10 min	
Final extension	68	10 min	1
Hold	10	Forever	

PrimeSTAR GXL DNA polymerase was selected as it consistently resulted in specific amplicons. Alternative high-fidelity DNA polymerases such as the KOD Hot Start DNA polymerase (#71086, Merck) can also be used.
  b. PCR products were separated and visualized on a 0.8% agarose gel containing RedSafe 1**×**, which is a substitute for ethidium bromide.
  c. **Optional (but recommended):** Add 1 μL of *Dpn*I restriction enzyme directly to the PCR mixture without prior treatment and incubate for 1 h at 37°C in a temperature-controlled heat block to digest template DNA. After 1 h, add another 1 μL of *Dpn*I and incubate for an extra hour.
  d. *Dpn*I-treated PCR products are purified using the High Pure PCR product purification kit (Roche) following manufacturer’s recommendations (here).
  e. Purified PCR products are quantified individually by measuring the absorbance at 260 nm using the μCuvette® G1.0 of the Biospectrophotomer (Roche) and concentrations are adjusted to 100 ng/μL when possible.

9. Yeast spheroplasts preparation
  a. Start an overnight culture of *S. cerevisiae* VL6-48N in 10 mL YPDA (pH 6.5) medium at 30°C under agitation (200 rpm).
  b. The next morning, measure the OD_600nm_ and use the appropriate volume to start a fresh 100-mL *S. cerevisiae* VL6-48N culture in YPDA with an initial OD_600nm_ of ∼0.2.
  c. When the OD_600nm_ of this new culture reaches ∼2, collect yeast cells by centrifugation at 1,750 **×** *g* for 3 min, discard the supernatant and resuspend the pellet in 20 mL of 1M sorbitol. Incubate overnight at 4°C.
  d. Collect the yeast cells by centrifugation at 1,750 **×** *g* for 3 min at 4°C. Resuspend the pellet in 10 mL of SPEM solution (see recipe) in a 50-mL sterile centrifuge tube.
  e. Add 20 μL of ß-mercaptoethanol and 20 μL of Zymolyase®-100T solution (see recipe) and incubate at 30°C with gentle agitation (80 rpm) until spheroplasts are ready.
    **CRITICAL:** The formation of spheroplasts needs to be assessed by comparing the OD_600nm_ measurements of a 1/10^th^ dilution of the spheroplast solution in a 1M sorbitol solution (intact spheroplasts) versus in a 2% SDS solution (lysed spheroplasts). Spheroplasts are considered ready when the ratio between the two readings is comprised between 3 and 4. Ideally, OD_600nm_ of 0.8 and 0.2 should be obtained when cells are diluted in 1M sorbitol and 2% SDS solutions, respectively.
  f. When spheroplasts are ready, add immediately 40 mL of 1M sorbitol and mix gently by inversion.
  g. Collect the yeast cells by centrifugation at 1,200 **×** *g* for 5 min at 4°C.
  h. Gently resuspend the pellet in 20 mL of 1M sorbitol using a 25-mL serological pipette. Add another 30 mL of 1M sorbitol and gently invert the tube 2–3 times.
  i. Collect spheroplasts by centrifugation at 1,200 **×** *g* for 5 min and resuspend the pellet in 2 mL STC (see recipe). Incubate at room temperature for 10 min.

10. Spheroplast transformation
  a. Mix 100 μL of yeast spheroplast with 5 μg of ASFV genomic DNA and 250 ng of the PCR-amplified pCC1BAC-Ura3 TAR vector containing the appropriate hooks (i.e., sharing overlaps with the ASFV sub-genomic fragment to clone). Incubate at room temperature for 10 min.
    ***Note:*** One transformation is performed per tube, which corresponds to the individual TAR cloning of one ASFV sub-genomic fragments. In total, seven independent transformations need to be performed in parallel in order to individually cloned all ASFV sub-genomic fragments described here.
  b. Add 0.5 mL of PEG solution (see recipe). Mix by rotating the tube very gently. Incubate at room temperature for 20 min.
  c. Collect the cells at 2,500 **×** *g* for 5 min. Carefully remove the supernatant and resuspend the pellet in 700 μL SOS solution (see recipe). Incubate for 2 h at 30°C.
  d. Mix the spheroplasts with 12 mL of melted TOP agar medium (see recipe), previously equilibrated at 55°C in a bead/water bath. Quickly pour the mixture onto Sorbitol-Ura plates (see recipe) and allow it to solidify for few seconds at room temperature.
  e. Incubate at 30°C for 2 days.
    ***Note:*** If no transformants are observed, see the troubleshooting section (problem 1).

### PCR-based screening of yeast transformants

**Timing: 2 days**

**Figure 2:**
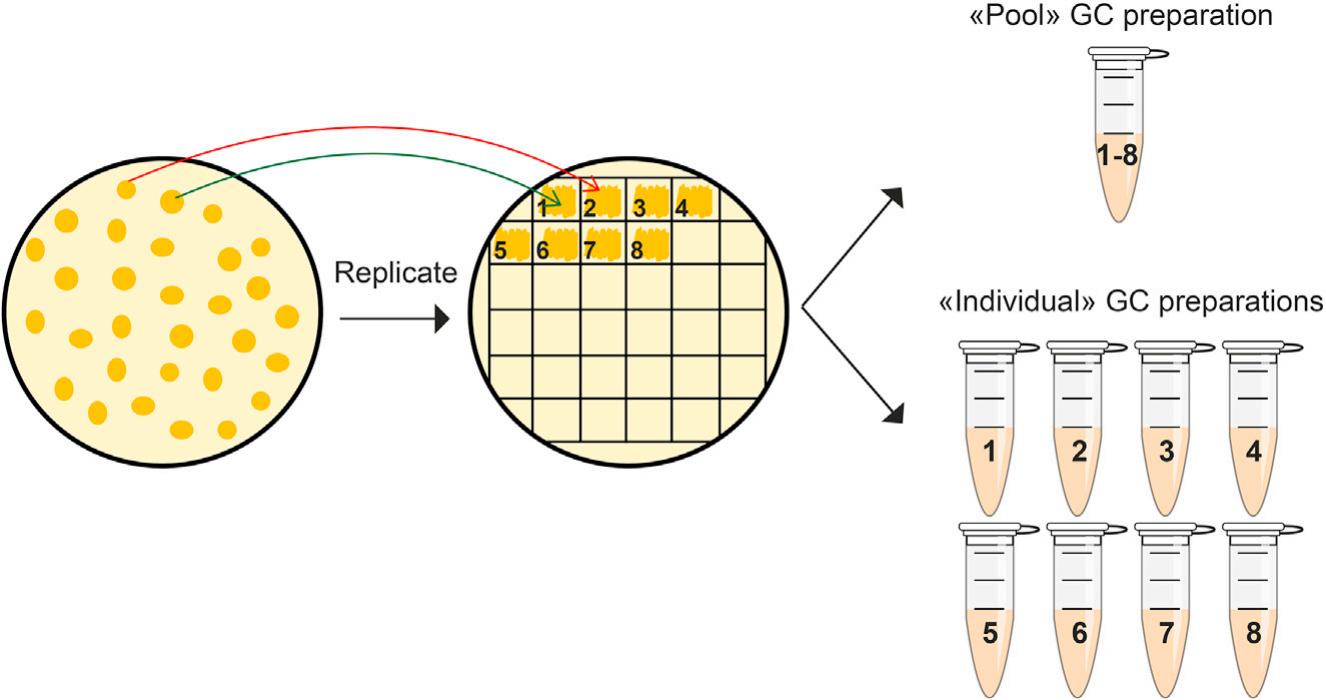
Flowchart describing the steps required for the PCR-based screening of yeast transformants Yeast colonies obtained on Sorb-Ura plates are replicated individually onto a fresh SD-Ura plates. A first GC extraction is performed on eight patches pooled together into a single tube (“Pool” GC preparation). New GC extractions are then carried out on each of the eight patches from a positive pool (“Individual” GC preparations). These steps are repeated for every TAR-cloned sub-genomic fragment.

This section describes the PCR-mediated identification of yeast transformants carrying the sub-genomic ASFV fragments of interest. Figure 2 summarizes the different steps involved in this section. The first step consists in the replication of individual yeast colonies on a new selection plate to ensure adequate propagation. The first screening relies on the amplification of a short internal region of the desired fragment, which is performed on pools of eight yeast colonies to allow a large but rapid initial pre-screening. The same PCR is then carried out on all individual colonies of previously tested positive pools to identify individual positive yeast clones. The second and third PCRs target the junctions between the TAR vector and the ASFV sub-genomic fragments.

**Figure 3:**
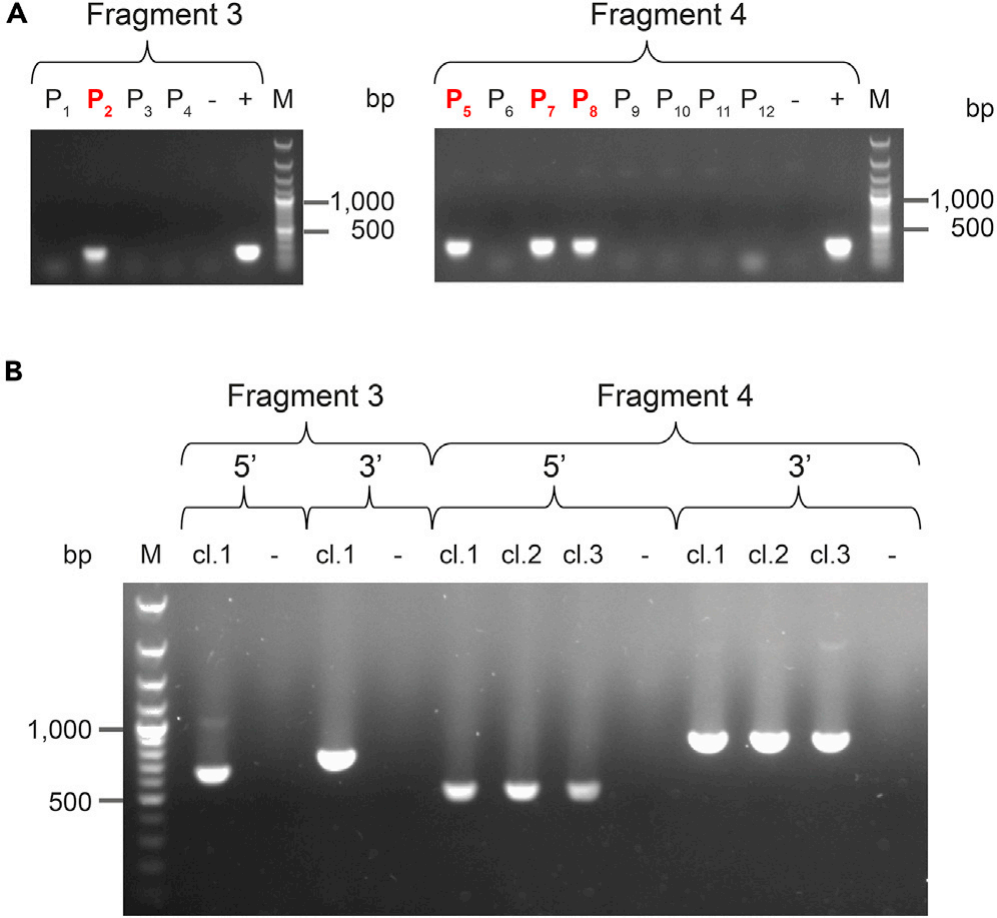
PCR-based screening and identification of yeast clones carrying an ASFV sub-genomic fragment (A) Agarose gels showing the amplification products of the internal DNA regions located on ASFV sub-genomic fragment 3 (left) and sub-genomic fragment 4 (right). PCRs were carried on pools (P) of eight colonies. Pools indicated in red are considered positives. (B) Amplification products obtained when the 5′ and 3′ junctions between the TAR vector and each of the two sub-genomic fragments were assessed. Plus and minus signs indicate the positive (ASFV gDNA) and negative (water) controls for each of the PCRs, respectively. GeneRuler 100 bp Plus DNA ladder (Thermo Scientific) was used as marker (M).

11. Pick isolated colonies from the original Sorbitol-Ura plates and patch each of them onto fresh SD-Ura plates. A total of 32 colonies are usually patched per plate using a 32-square grid PetriSticker™ (Sigma-Aldrich). Incubate at 30°C for 24 h.
12. Extract yeast (extra)-chromosomal DNA using the glass bead Chelex 100 preparation (GC prep) method as described elsewhere (Blount et al., 2016).
  a. Collect a ∼2-mm^2^ surface of a yeast patch with the tip of a 20-uL sterile pipette tip and placed into an 1.5mL-Eppendorf tube containing 100 μL of a 5% Chelex 100 solution (resuspended in deionized water) and add acid washed glass beads to half total sample volume.
  b. Repeat step a) for 7 additional patches (in order to make a pool of 8 colonies)
  c. Vortex at 1,400 rpm for 4 min.
  d. Incubate for 2 min at 99°C and centrifuge at 18,000 **×** *g* for 1 min.
  e. Transfer 30–40 μL of the supernatant to a clean tube. Store at 4°C for 24–48 h or at −20°C for a longer period.
  f. Use 1 μL of the supernatant as DNA template in each of the subsequent screening PCRs

13. PCR amplifications of i) the internal genomic DNA region and ii) the two junctions between the TAR vector and ASFV gDNA fragment are performed. Amplicons obtained from pooled clones and individual clones are displayed in Figure 3.
  a. PCR reactions are performed using the GoTaq® G2 Green Master mix (Promega) according to manufacturer’s recommendations (see here). The set-up for each reaction is presented in the following table:

**Table:** 

Set-up PCR reaction (for one reaction) (GoTaq G2 green master mix)	
GoTaq® G2 Green Master Mix, 2**×**	12.5μL
upstream primer, 10μM	2 μL
downstream primer, 10μM	2 μL
DNA template (GC prep, step 12)	1 μL
Nuclease-Free Water	7.5 μL
Total	25 μL
  b. PCRs are run following the cycling conditions described below:

**Table:** 

PCR cycling conditions (GoTaq G2 green master mix)			
Steps	Temperature	Time	Cycles
Initial denaturation	95	2 min	1
Denaturation	95	15 s	30
Annealing	50	30 s	
Extension	72	1 min	
Final extension	72	5 min	1
Hold	10	Forever

    ***Optional:*** Sanger sequencing of the different PCR products is recommended to confirm the identity of the ASFV sub-genomic fragment cloned and that the junctions do not contain any SNPs that would be detrimental for the subsequent steps.

14. Select one or two yeast clones containing the desired fragment for each of the five TAR cloning experiments for further use. Save these yeast clones at −80°C in Roti®Store yeast cryo vials following manufacturer’s conditions (see here).

If no positive yeast transformants are obtained, see troubleshooting section (problem 2).

### Multicopy plasmid induction in *E. coli*

**Timing: 2 days**

This section describes the extraction of the yeast artificial chromosomes (YACs) containing ASFV sub-genomic fragments from yeast followed by their subsequent transformation into *E. coli*. The multicopy induction system present on the pCC1BAC-Ura3 TAR vector is then used to purify larger amounts of plasmids/YACs required for the final reassembly in yeast.

15. YAC extraction from yeast based on the QIAGEN mini kit
  a. Resuspend ∼half of the remaining yeast patch from positive clones (from step 16) into 250 μL of P1 solution (included in the QIAGEN mini kit), 25 μL of Zymolyase solution (see recipe) and 2.5 μL of β-mercaptoethanol. Vortex for 10 s and incubate the mixture at 37°C for 30 min.
  b. Vortex for another 10 s and incubate for 30 min at 37°C.
  c. Add 250 μL of P2 solution and follow all the next steps according to manufacturer’s instructions (here) up to the elution step.
  d. For the elution step, add 30 μL of elution buffer into the column and let sit for 5 min. Place the column into a fresh microcentrifuge tube and centrifuge for 1 min at 18,000 **×** *g* at room temperature.
16. *E. coli* transformation using Transformax™ EPI300™ electrocompetent cells
  a. Chill 1-mm electroporation cuvettes and sterile Eppendorf tubes on ice for 15 min (one cuvette and one tube per transformation).
  b. Add 3 μL of the previously extracted plasmid solution to 35 μL of freshly thawed Transformax™ EPI300™ electrocompetent *E. coli* cells in a pre-chilled tube.
  c. Transfer the mixture to the pre-chilled 1-mm electroporation cuvette and electroporate following manufacturer’s instructions. In our case, we used the Gene Pulser Electroporation system (Bio-Rad) with the following parameters: 2 kV; 25 μFD and 200 Ohms.
  d. Add immediately 950 μL of SOC medium and incubate for 1 h at 37°C under agitation (200 rpm).
  e. Transfer the mixture to a fresh sterile microcentrifuge tube and centrifuge for 30 s at 18,000 **×** *g*, discard the supernatant.
  f. Resuspend the pellet in 200 μL of fresh SOC medium and plate on selective medium (LB-Agar plates containing 12.5 μg/mL chloramphenicol). Transformants are usually observed after 24 h of incubation at 37°C.

***Note:*** An additional 24 hours incubation might ease the picking of the *E. coli* colonies in some cases.

17. PCR verification of the *E. coli* clones.
  a. Resuspend one *E. coli* colony per ASFV sub-genomic fragment in a fresh microcentrifuge tube containing 10 μL of deionized water.
  b. Use 1 μL of the mixture as DNA template for each of the two junction PCRs as previously described in step 18.
  c. Use the remaining 8 μL to start a 12-mL culture in LB-Chl^R^_12.5_ for each positive *E. coli* clone tested. Incubate for 12–14 h at 37°C under agitation (220 rpm).
  d. Save one or two *E. coli* clones at −80°C using 750 μL of the overnight cultures mixed with glycerol (15% final concentration).

18. Multicopy induction and plasmid purification from *E. coli* clones
  a. Use the remaining 10 mL of the overnight culture (from step 22) to start a 100-mL LB-Chl^R^_12.5_ culture into a 250-mL Erlenmeyer baffled flask to ensure proper aeration.
  b. Add 100 μL of CopyControl™ induction solution to the culture and incubate for 5 h at 37°C under agitation (220 rpm).
    **CRITICAL:** Proper aeration of the culture is extremely important to ensure high yields of plasmid after purification.
  c. Centrifuge the culture for 20 min at 4,250 **×** *g* at room temperature. Pellets can be stored at −20°C.
  d. Plasmids were extracted using the QIAGEN midiprep plasmid purification kit following manufacturer’s instructions (here).
  e. Quantify DNA concentration using a biospectrophotometer.

### Full-length reassembly of ASFV clones

**Timing: 1 day (+2 days after yeast transformation)**

This section describes the reconstruction of full-length ASFV genomes in yeast. It consists in the co-transformation of *S. cerevisiae* with a mixture of precipitated DNA containing all five overlapping ASFV sub-genomic fragments as well as the two chemically-synthetized fragments 1 and 2. This section also contains the final screening step of the reconstructed ASFV genomes confirming the presence of all the junctions between the seven different ASFV sub-genomic fragments.

**Figure 4:**
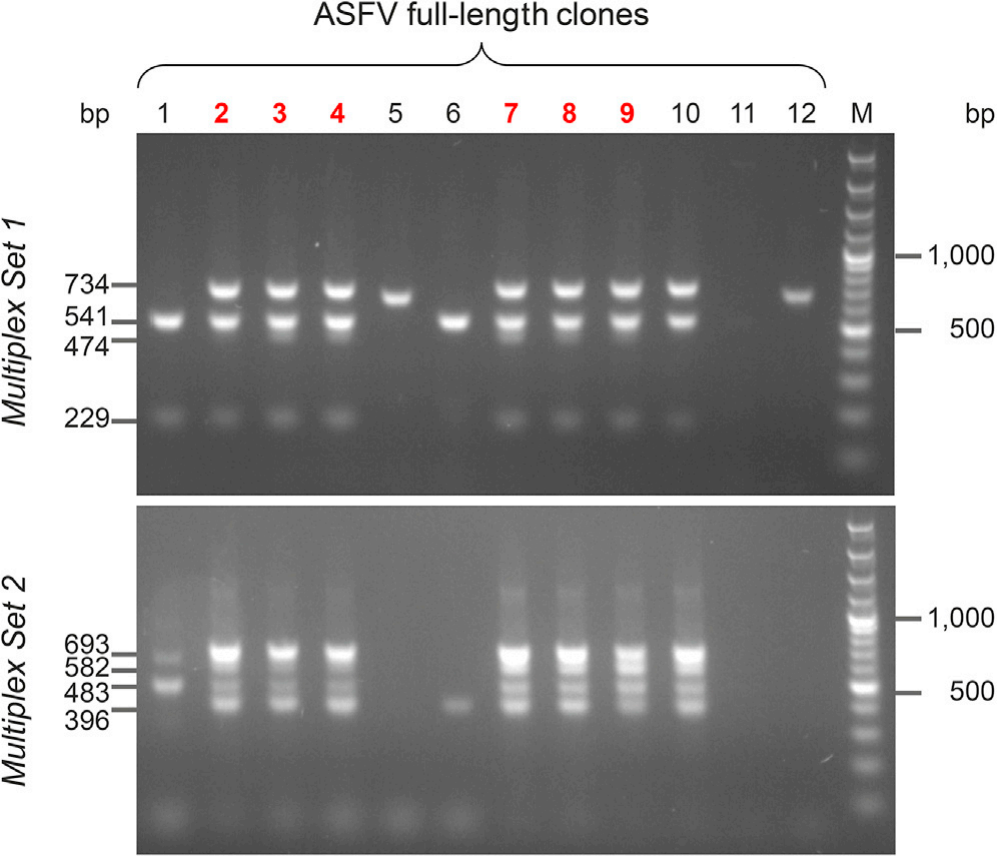
Identification of positive full-length clones using multiplex PCRs screening Two independent multiplex PCRs were performed on full-length assembled ASFV constructs with two sets of primers in order to ensure the presence of all junctions between the different ASFV sub-genomic fragments. Amplification profiles expected for the multiplex Set1 (top) and multiplex Set2 (bottom) can be observed for positive clones (in red). Expected sizes of the PCR products are indicated on the left side of the gels. GeneRuler 100 bp Plus DNA ladder (Thermo Scientific) was used as marker (M).
  d. Cycling conditions for the two PCRs are described in the table below:

19. Gel purification of the two synthetic fragments 1 and 2
  a. Five micrograms of the pUC57-Fragment1 plasmid were *Sma*I-digested in five 50-μL total reaction volume each containing 1 μg of DNA. Digestions were performed following manufacturer’s instructions (NEB). The same protocol was applied to the digestion of the pUC57-Fragment2 plasmid.
  b. Digested products were loaded on a 1% TAE agarose gel containing RedSafe™ 1**×**. Electrophoresis was carried out for 1 h at 50 V (3.85 V/cm).
  c. The DNA bands corresponding to the linearized synthetic constructs were excised from the gel using an X-tracta gel extractor tool (Sigma-Aldrich) after short exposure to blue light using an LED transilluminator (MaestroGen).
    ***Note:*** Blue light should be preferred over ultraviolet light to avoid DNA damage during the purification process
  d. The excised gel band was placed in a 1.5-mL microcentrifuge tube and DNA was eluted in 30 μL final volume using the QIAQuick gel extraction kit following manufacturer’s instructions (QIAGEN).
  e. Quantify DNA concentrations using a biospectrophotometer. One hundred fmoles of each of the *Sma*I-digested Fragment1 and Fragment2 are used for step 26.

20. Linearization of the YACs containing the different ASFV sub-genomic fragments
  a. Digest 50 fmoles of each of the ASFV sub-genomic fragments individually by I-*Sce*I following manufacturer’s instructions (here). If DNA concentration is too low, multiple 50-μL reactions can be performed in parallel.
  b. After 1 h restriction time, add 1 μL of I-*Sce*I to the reaction and incubate for an extra hour.
21. Precipitation of DNA prior yeast transformation
  a. All digested ASFV fragments (synthetic and TAR-cloned ones) are pooled together in a microcentrifuge tube.
  b. The total volume (V_DNA_) of all digested ASFV fragments is calculated. Digested ASFV fragments is precipitated in a solution containing 1/10^th^ V_DNA_ of isopropanol; 1/10^th^ V_DNA_ of 3M NaAc; 1 μL of Glycoblue™ coprecipitant (ThermoFisher) and 1 μL of tRNA (Sigma-Aldrich).
  c. Centrifuge the mixture at 12,500 **×** *g* for 30 min at 4°C and discard the supernatant.
  d. Resuspend the pellet in 700 μL of 70% ethanol solution.
  e. Centrifuge at 12,500 **×** *g* for 15 min at 4°C and discard the supernatant.
  f. Resuspend the pellet in 30 μL of TE buffer (10 mM Tris-HCl pH8; 1 mM EDTA). Store at 4°C if used immediately or at −20°C for a longer period.
22. Yeast spheroplast co-transformation with all ASFV sub-genomic fragments and final pCC1BAC-His3 TAR vector
  a. Yeast spheroplasts were prepared as previously described (step 14).
  b. Mix yeast spheroplast with 250 ng of PCR-amplified pCC1BAC-His3 and the 30 μL of all seven precipitated ASFV fragments.
  c. The following steps were performed as previously described in step 15 with the exception that yeast cells were plated on Sorbitol-His plates instead of Sorbitol-Ura. Transformants can be observed after 24 h and colonies are big enough to be picked after 48 h.

23. Screening of yeast transformants using multiplex PCR
  a. Patch yeast colonies onto a fresh SD-His plate.
  b. Extract yeast DNA using the GC-prep method as previously described (step 17)
  c. Use 1 μL as DNA template and set up two independent multiplex PCR reactions for individual colonies using the Qiagen® Multiplex PCR kit (Qiagen) following manufacturer’s instructions (here). Expected profiles for ASFV full-length clones are presented in Figure 4. All primers included in each multiplex PCR can be found in the primer table here. The set-up for each reaction is presented in the following table:

**Table:** 

Set-up PCR reaction (for one reaction) (multiplex PCR kit Qiagen)	
2**×** QIAGEN Multiplex PCR Master Mix	12.5 μL
10**×** primer mix, 2 μM each primer	2.5 μL
RNase-free water	9 μL
DNA template (GC prep, step 23b)	1 μL
Total	25 μL

**Table:** 

PCR cycling conditions			
Steps	Temperature	Time	Cycles
Initial denaturation	95	15 min	1
Denaturation	95	1 min	35
Annealing	50	2.5 min	
Extension	72	1.5 min	
Final extension	68	10 min	1
Hold	10	Forever

24. Select one or two yeast positive transformants for further use. Biobank these yeast clones at −80°C in Roti®Store yeast cryo vials following manufacturer’s conditions (see here).

If no positive yeast transformants are obtained, see troubleshooting section (problem 5).

25. Large-scale preparation of full-length ASFV genomic DNA
  a. YAC extraction from yeast
    i. Start a 500-mL culture of *S. cerevisiae* containing full-length ASFV construct in SD-His at 30°C under agitation in a 2 L baffled Erlenmeyer flask.
    ii. When OD_600nm_ reaches ∼2, centrifuge the culture at 4,250 **×** *g* for 20 min at 4°C.
    iii. Resuspend the pellet in a solution containing 20 mL of P1 solution (included in the QIAGEN® Large-construct kit), 2 mL of Zymolyase solution (see recipe) and 200 μL of β-mercaptoethanol. Vortex for 30 s and incubate the mixture at 37°C for 1 h.
    iv. Vortex for another 30 s and incubate for another 1 h min at 37°C.
    v. DNA extraction is performed using the Qiagen® Large-construct kit following manufacturer’s instructions (here)
  b. Plasmid extraction from *E. coli*
    i. Repeat steps 20 and 21 with an *S. cerevisiae* clone carrying a full-length ASFV construct.
    ii. Proceed to DNA extraction using the Qiagen® Large-construct kit starting with a 300-mL *E. coli* culture following manufacturer’s instructions (here).

***Note:*** Alternatively, agarose plugs containing intact ASFV chromosome-sized DNA can be prepared following manufacturer’s instructions (here). Such plugs can be prepared from either *E. coli* or yeast cultures using the CHEF Bacterial Genomic DNA plug kit (Bio-Rad, 1703592) or CHEF Yeast Genomic DNA plug kit (Bio-Rad, 1703593), respectively.

### Genetic engineering of ASFV clones

**Timing: 3 days**

**Figure 5:**
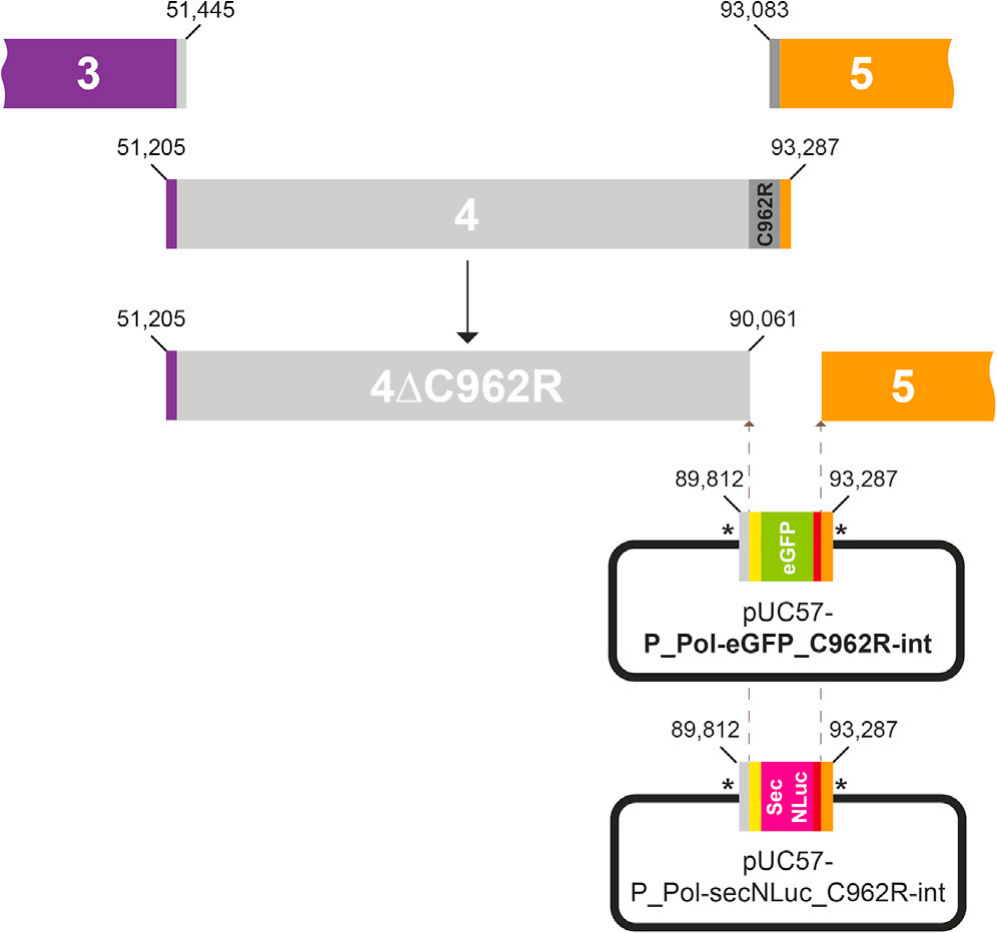
Design for the replacement of the ASFV C962R gene with synthetic DNA cassettes carrying the eGFP or secNLuc reporter genes The ASFV sub-genomic fragment 4 excluding the C962R gene (Fragment 4ΔC962R) was first isolated using TAR cloning. Two DNA cassettes, namely the P_Pol-eGFP_C962R-int or P_Pol-secNLuc_C962R-int, were chemically synthesized and cloned in the pUC57 plasmid. They carry either the eGFP and secreted Luciferease reporter genes and contain overlaps with their neighboring fragments during genome reassembly, namely Fragment 4ΔC962R and Fragment 5. Asterisks indicate the presence of *Sma*I restriction sites used for linearization. All genetic features included in the synthetic DNA cassettes are detailed in step 31.

This section describes the necessary steps for the engineering of the ASFV genome using the previously isolated sub-genomic fragments. Here, we substituted one non-essential gene in Fragment 4 (namely the C962R gene; Figure 5, in dark gray) (Ramirez-Medina et al., 2020) and replaced it by chemically-synthetized DNA cassettes carrying reporter genes as a proof of concept.

26. Design of the chemically-synthetized DNA cassettes carrying the reporter genes
  a. ***Sma*I restrictions sites (indicated with asterisks in**Figure 5**):** Two *Sma*I restrictions sites were added at the 5′ and 3′ ends of the DNA cassettes to ensure linearization and purification from the pUC57 backbone plasmid.
  b. **5′-overlap (**Figure 5**, in light gray):** a region of 250 bp, overlapping the ASFV genome upstream of the C962R coding sequence included in the ASFV sub-genomic fragment 4, was added to ensure legitimate recombination in yeast.
  c. **Promoter region (**Figure 5**, in yellow):** the promoter region of the DNA polymerase (G1211R) of the ASFV Armenia isolate was used as previously reported (Portugal et al., 2017).
  d. **Reporter sequences:** the respective 720-bp and 600-bp nucleotide sequence of the enhanced GFP (eGFP) (Figure 5, in green) and secreted Luciferase (secNLuc) (Figure5, in pink) obtained from Promega were added in frame with the promoter region.
  e. **Terminator sequence (**Figure 5**, in red):** the 281-bp terminator sequence of the thymidine kinase (TK, L60) was added after the reporter sequences in both constructs (Portugal et al., 2017).
  f. **3′-overlap (**Figure 5**, in orange):** a 387-bp region consisting of the last 65 bp of the 3′UTR of the C962R open reading frame (Cackett et al., 2020). The rest of the sequence, overlapping with the ASFV sub-genomic fragment 5, was added to ensure legitimate recombination in yeast.
27. Isolation of the ASFV sub-genomic fragment 4 deleted for the C962R gene
  a. Generation of pCC1BAC-Ura3 with appropriate hooks. A PCR amplification of the pCC1BAC-Ura3 TAR vector was carried out with primers ASFTARhook4-R/F2 as reported in step 8.
  b. Yeast clones carrying the ASFV sub-genomic fragment 4 deleted for C962R were obtained using TAR cloning as reported in steps 9 and 10.
  c. Repeat steps 11 to 18 in order to obtain purified YACs containing the ASFV sub-genomic fragment 4 deleted for C962R.
28. Full-length reassembly of genetically engineered ASFV genomes carrying eGFP or secNLuc reporter genes
  a. Repeat steps 24 to 30 including one of the two chemically-synthetized DNA cassettes, namely pUC57-P_Pol-secNluc _C962R-int or pUC57-P_Pol-eGFP_C962R-int, previously gel purified along with all the required ASFV sub-genomic fragments.

## Expected outcomes

The main outcome of this protocol is to provide a fast and flexible genetic engineering platform for the modification of genomes derived from large DNA viruses, such as the ASFV, to the scientific community. All full-length constructs (wild-type full-length ASFV construct and its respective eGFP and secNLuc versions), as well as all the individual ASFV sub-genomic fragments described in the protocol will be made available to the scientific community upon request.

## Limitations

One current limitation of the system is the need to clone all sub-genomic fragments in *E. coli* prior to the final reassembly in yeast. This is due to the difficulties encountered when large YACs have to be purified directly from *S. cerevisiae*. Indeed, YACs such as the ones described here are maintained as single copy plasmid in yeast. Along with the purification issues due to yeast genomic contamination, it is technically challenging to produce large quantities of pure YACs from yeast cultures. Propagation of the constructs in *E. coli* can be associated with SNPs introductions or stability issues. Efforts are ongoing to circumvent these difficulties so that a “yeast-only” system can be achieved.

## Troubleshooting

### Problem 1

No colonies are obtained after yeast spheroplast transformation (step 15)

### Potential solutions

Make sure that the ratio observed in step 9.e during spheroplast formation is correct. A significantly different ratio will affect the competency of the yeast preparation. If needed, a second culture can be started and used as backup in case the first culture fails to provide the appropriate ratio.

Check that the proper auxotrophic marker was used to prepare the agar plates.

Ensure that the PEG solution has been freshly prepared and that the pH of the all solutions is adequate.

The amount of DNA to use for the yeast transformation should not exceed 10%–20% of the total reaction volume. A large DNA volume will negatively impact the transformation efficiency.

### Problem 2

No positive yeast clones obtained after the PCR-based screening (step 18)

### Potential solutions

Always confirm that the TAR vector has been specifically amplified. Always include a negative control in each transformation experiment consisting of the PCR-amplified TAR vector only. If a high number of colonies is present on the negative control plate, consider doing another round of *Dpn*I treatment or gel-purify your TAR vector before use.

Ensure that the primers used to PCR amplify the TAR vector have been designed correctly. As these primers are typically ∼80-nt long, it is generally recommended to add an extra PAGE purification step after synthesis.

The sequences used for the overlapping regions should be unique and should not consist of highly repetitive regions that can trigger illegitimate recombination during TAR cloning.

Check the quality of the purified viral DNA. This is of great importance, especially when large sub-genomic DNA fragments are to be TAR-cloned.

Ensure that the GC extractions as well as the designed primers do not alter the amplification of the targeted internal regions by running appropriate PCR controls including a positive one using ASFV genomic DNA as template.

### Problem 3

Low concentration of DNA after induction in *E. coli* (step 23)

### Potential solutions

Make sure that the aeration of the *E. coli* culture is optimal. To do so, make sure to use baffled Erlenmeyer flasks able to contain at least 5 times the volume of culture actually used.

### Problem 4

Incorrect DNA profiles after I-*Sce*I digestion (step 25)

### Potential solutions

Check for the presence and correct sequence of each the I-*Sce*I restriction sites by Sanger sequencing after PCR amplification. If problems are observed, use a different *E. coli* clone.

**Figure 6:**
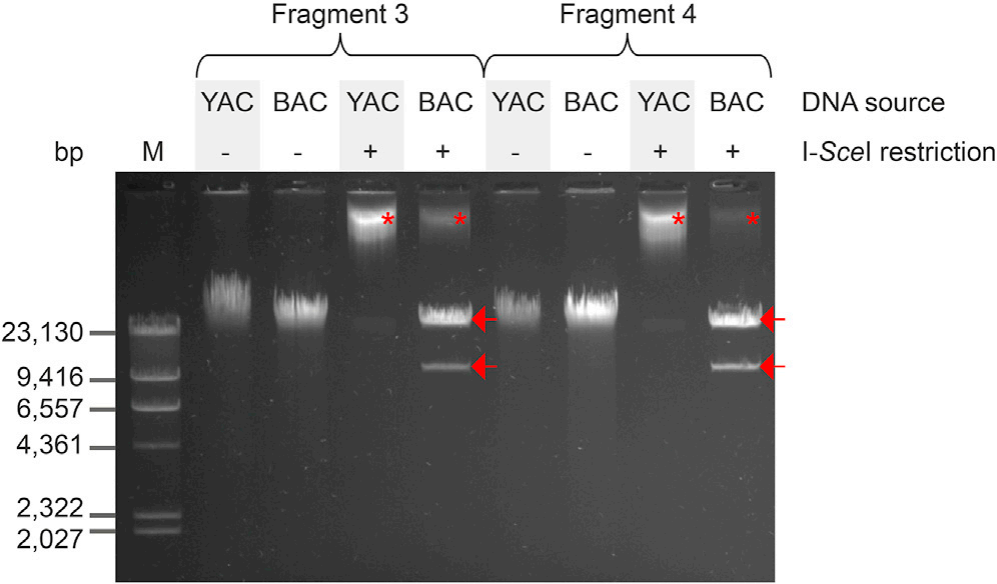
Restrictions profiles of ASFV subgenomic fragments extracted from yeast or *E. coli* Agarose gel showing plasmid DNA extracted from *S. cerevisiae* (YAC) or *E. coli* (BAC) undigested (-) or digested with I-*Sce*I (+) for two different ASFV sub-genomic fragments. Red asterisks indicated the presence of genomic DNA in the plasmid extractions, which is particularly present and problematic when plasmids are extracted from yeast cultures. The expected digestion pattern is shown by the two red arrows corresponding to the linearized ASFV sub-genomic fragments (top) and TAR vector (bottom), respectively, and only obtained with *E. coli* derived plasmid DNA. The Lambda DNA/HindIII ladder (ThermoFischer) (Thermo Scientific) was used as marker (M).

Determine the presence of possible genomic DNA contamination in your samples by loading each of them on a 0.8% agarose gel (as shown in Figure 6). Genomic DNA contamination might artificially decrease the concentration of plasmid DNA in your samples. If so, start a new plasmid DNA preparation and reduce the initial volume of the culture to 50 mL.

Mechanical shearing might be observed on a 0.8% agarose gel as a DNA smear. Make sure to slowly and carefully mix your plasmid DNA preparations by pipetting at little as possible or use wide-bore tips if necessary.

### Problem 5

No positive full-length clones after screening using the multiplex PCRs (step 28)

### Potential solutions

Make sure that all the PCRs included in the multiplex PCR reactions are working when performed individually using the genomic DNA as DNA template.

Make sure that each sub-genomic fragment overlap its two neighboring fragments by at least 50 bp.

Make sure that all sub-genomic fragments were included in the pool of DNA transformed in yeast.

## Resource availability

### Lead contact

Further information and requests for resources and reagents should be directed to Dr. Fabien Labroussaa. Email: fabien.labroussaa@vetsuisse.unibe.ch

### Materials availability

All plasmids and resources generated during this study will be made available upon request. Recipients will be asked for proofs concerning their capacities to work with BSL-2 and BSL-3 agents before any shipment of material.

## Data Availability

The genomic sequence of the abovementioned ASFV isolate was determined using Illumina sequencing performed at the Next-Generation Sequencing platform (University of Bern). The sequence is identical to the isolate Georgia 2007/1 (GenBank: FR682468.2) previously published (Chapman et al., 2011). This protocol does not report original code.
